# Chondromatose du genou compliquée d'un genou valgum secondaire avec compression de nerf sciatique poplité externe

**DOI:** 10.11604/pamj.2016.23.127.8714

**Published:** 2016-03-25

**Authors:** Mohamed Amine karabila, Ahmed Bardouni

**Affiliations:** 1 Service de Chirurgie Traumato-orthopédie, CHU Ibn Sina, Rabat, Maroc

**Keywords:** Chonromatose, genou, sciatique, Chondromatosis, knee, sciatica

## Image en médecine

La chondromatose est une arthropathie chronique rare caractérisée par une métaplasie de la synoviale aboutissant à la formation de corps ostéocartilagineux dans une cavité articulaire. Nous présentons un cas très rare d'un chondrome géant comprimant le nerf sciatique poplité externe et se compliquant d'un genou valgum avec dégradation cartilagineuse du compartiment externe. Nous rapportons l'observation d'un homme de 25 ans consultant pour une tuméfaction du genou depuis 2 ans. L'examen a objectivé trois masses mobiles au niveau de son genou, une raideur estimée à 10° d'extension et 110° de flexion et des paresthésies surtout nocturnes au niveau de la face dorsale du pied sans déficit moteur et un genou valgum droit manifeste estimé à 12°. Les radiographies du genou (A et B) et l'IRM (C) ont objectivés trois formations osseuses radio-opaques avec un grand ostéophyte postérieure du condyle fémorale externe. L'exploration chirurgicale a mis en évidence une compression partielle du nerf sciatique poplité externe (D) par l'une de ces formations qui loge au niveau de la face externe du genou puis nous avons procédé à une ablation de toutes les masses cartilagineuses visibles (E).

**Figure 1:**
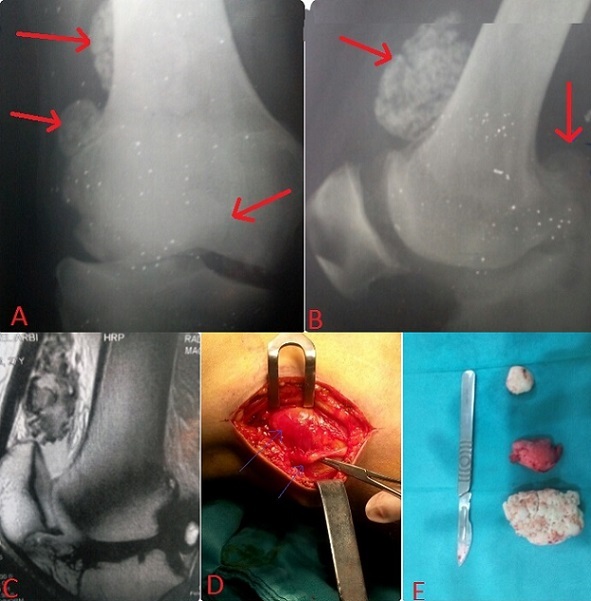
A et B) radiographies du genou montrant les ostéochondromes multiples; C) IRM du genou confirmant la nature ostéocartilagineuse de ces lésions; D) image opératoire montrant la compression du nerf par la masse tumorale; E) aspect macroscopique des ostéochondromes

